# Hexavalent Chromium Oropharyngeal Aspiration Induced Behavior Effects and Essential Metal Dyshomeostasis in Young Hartley Guinea Pigs

**DOI:** 10.3390/app16010059

**Published:** 2025-12-20

**Authors:** Samuel T. Vielee, Idoia Meaza, William J. Buchanan, Spencer H. Roof, Haiyan Lu, Sandra S. Diven, Luping Guo, Jack Easley, J. Calvin Kouokam, Jamie Lynn Wise, Aggie R. Brownell, John Pierce Wise, John P. Wise

**Affiliations:** 1 Department of Pediatrics, University of Louisville, Louisville, KY 40292, USA; 2 Department of Pharmacology and Toxicology, University of Louisville, Louisville, KY 40292, USA; 3 Wise Laboratory of Environmental and Genetic Toxicology, University of Louisville, 500 S Preston Street, Building 55A, Room 1422, Louisville, KY 40292, USA; 4 Department of Pharmacology and Experimental Therapeutics, University of Toledo, 3100 Transverse Dr., Health Education Building Room 282C, Toledo, OH 43614, USA

**Keywords:** hexavalent chromium, guinea pigs, neurotoxicity, behavior effects, inhalation exposure, sex differences, metallomics

## Abstract

Hexavalent chromium [Cr(VI)] is the toxic form of chromium often used in industry for its hardness, bright colors, and anticorrosive properties. Cr(VI) is a known human lung carcinogen, making its inhalation an occupational hazard. Growing evidence emphasizes the neurotoxic potential of Cr(VI), though it is not linked to brain cancers. Few studies consider neurotoxicity in chromate workers, reporting impaired olfactory discrimination and an increased risk of death from mental health disorders. A major factor limiting translation of most rodent Cr(VI) studies to human populations has to do with vitamin C, which can reduce the toxic Cr(VI) to non-toxic Cr(III). Rats and mice synthesize vitamin C and are likely more resistant to Cr(VI) than humans. Here, we considered Cr(VI) neurotoxicity in guinea pigs (*Cavia porcellus*), which do not endogenously synthesize vitamin C. We exposed Hartley guinea pigs (both sexes) to occupationally relevant concentrations of Cr(VI) via oropharyngeal aspiration weekly for 90 days. We observed behavioral effects in the open field assay, elevated plus maze, Y-maze, and novel object recognition test during weeks 9–12 of exposure. After euthanasia, we assessed Cr accumulation and essential metal dyshomeostasis in the hippocampus. We observed significantly increased hippocampal Cr accumulation in females, while males exhibited essential metal dyshomeostasis.

## 1. Introduction

Chromium is a naturally occurring metal known to exist in two biologically relevant valence states—trivalent chromium [Cr(III)] and hexavalent chromium [Cr(VI)]. Until recently, Cr(III) was considered an essential metal, and many still believe it has pharmacological properties [1,2]. On the other hand, Cr(VI) is highly toxic and a known human lung carcinogen, with toxicity reported across organ systems—including the lungs, liver, kidneys, reproductive organs, and brain [1,3–5]. The underlying explanation is a difference in uptake. Cr(VI) forms chromate, which structurally resembles phosphate and sulfate anions. This structural mimicry allows chromate anions to rapidly enter cells through sulfate and phosphate anion transporters. Cr(III) binds to various ligands, resulting in a bulky structure that is poorly absorbed and limits its cellular uptake to slow passive diffusion. Thus, knowledge gathered from the Cr(III) literature cannot be directly extrapolated to Cr(VI). Drawing from its broad, but potent, toxicity, the Agency for Toxic Substances and Disease Registry’s Substance Priority List ranks Cr(VI) as the 17th greatest potential chemical threat to human health [6]. Despite the hazards of Cr(VI), it is used extensively in industry for its hardness, bright colors, anticorrosive properties, and electrical conductance [1]. With over 200 industrial applications, Cr(VI) human exposures, from occupational and environmental settings, remain a major public health concern.

Links between lung cancer and Cr(VI) occupational exposure were first reported nearly 200 years ago, but many knowledge gaps remain for other organ systems [7]. Neurotoxicity of Cr(VI) from occupational exposure is extremely limited in scope, with only three studies considering neurological effects in chromate workers. Two of these studies reported impaired olfactory discrimination in chromate production workers and chrome plating workers, unrelated to olfactory detection, while the third study reported increased risk of alcoholism [8–10]. Rodent and cell culture studies provide further insight into Cr(VI) neurotoxicity, though models mimicking occupational exposures are scarce. At least two groups assessed Cr(VI) neurotoxicity following intranasal instillation, reporting behavioral effects, oxidative stress, and inflammation [11,12]. These studies reported Cr(VI) induced locomotor impairment, but Hegazy et al. also reported changes in working memory [11].

Considering non-occupationally exposed individuals, we see more general characteristics of Cr(VI) neurotoxicity. In human populations, we can identify a link between age and brain Cr accumulation, with older individuals exhibiting higher brain Cr levels [5]. Epidemiological data and case studies link Cr(VI) to a host of neurological effects, including motor neuron disease, autism spectrum disorder, memory impairments, polyneuropathy, and acute or recently diagnosed schizophrenia [5,13–17]. Recent studies in rodents demonstrated Cr accumulation in the rodent brain after 14–28 days of daily intraperitoneal exposure to Cr(VI), with the hypothalamus accumulating the highest levels [18,19]. We recently reported exposure to environmentally relevant levels of Cr(VI) in drinking water induced behavior effects, Cr accumulation in the hippocampus, and essential metals dyshomeostasis in rats—likely a contributing factor for Cr(VI) neuropathogenesis [20–22]. Regardless of exposure route, Cr(VI) neurotoxicity manifests across multiple taxa, with behavior effects, oxidative damage, inflammation, and neurodegeneration frequently reported [5,12,20–26]. Locomotor dysfunction or decreased motor activity are most commonly reported, but a few other behavioral effects have been observed, including memory and anxiety [5,11,12,21,22,26]. One study exposed female mice to tannery effluent containing high levels of Cr(VI) and observed impaired social memory [23]. Notably, Estrela et al. found co-treatment with vitamin C alleviated these effects, linking the impaired memory to Cr(VI) exposure.

Vitamin C production is a key consideration for Cr(VI) toxicity studies, as most rodents endogenously produce vitamin C while humans do not. Guinea pigs (*Cavia porcellus*) are the only known rodent species that do not endogenously synthesize vitamin C, making them a useful model to assess Cr(VI) toxicity. Despite the relevance of guinea pig models to human health, guinea pigs are an underutilized model, with most Cr(VI) toxicity studies limited to dermal effects [27]. Here, we aimed to create a guinea pig model for assessing Cr(VI) neurotoxicity following occupationally relevant exposures. We exposed Hartley guinea pigs (both sexes) to Cr(VI) via oropharyngeal aspiration once per week for 90 days. We assessed behavior changes (locomotor function, anxiety, spatial memory, working memory), hippocampal Cr accumulation, and hippocampal essential metal homeostasis following exposure to 0.2, 0.4, or 0.8 mg/kg zinc chromate, compared to a saline-exposed control group.

## 2. Materials and Methods

### 2.1. Animals

Forty male and forty female Hartley guinea pigs, aged 63–69 days, were purchased from Charles River (Wilmington, MA, USA) and acclimated for one week prior to the start of the study. Guinea pigs were housed two animals per cage and kept on a 12 h light/dark cycle. Guinea pigs were provided 2040C Teklad Global Guinea Pig Diet (Envigo RMS Division, Indianapolis, IN, USA) and water ad libitum. Notably, this diet contains 1050 mg of vitamin C/kg and provides a daily consumption of approximately 21 mg of vitamin C per day. After acclimatization, microchips were subcutaneously implanted in all guinea pigs for identification (UC-2112–24, Unified Information Devices, Inc., Lake Villa, IL, USA). All animal studies were approved by the University of Louisville Institutional Animal Care and Use Committee (IACUC Protocol #23244).

### 2.2. Cr(VI) Exposure

**Figure 1.**
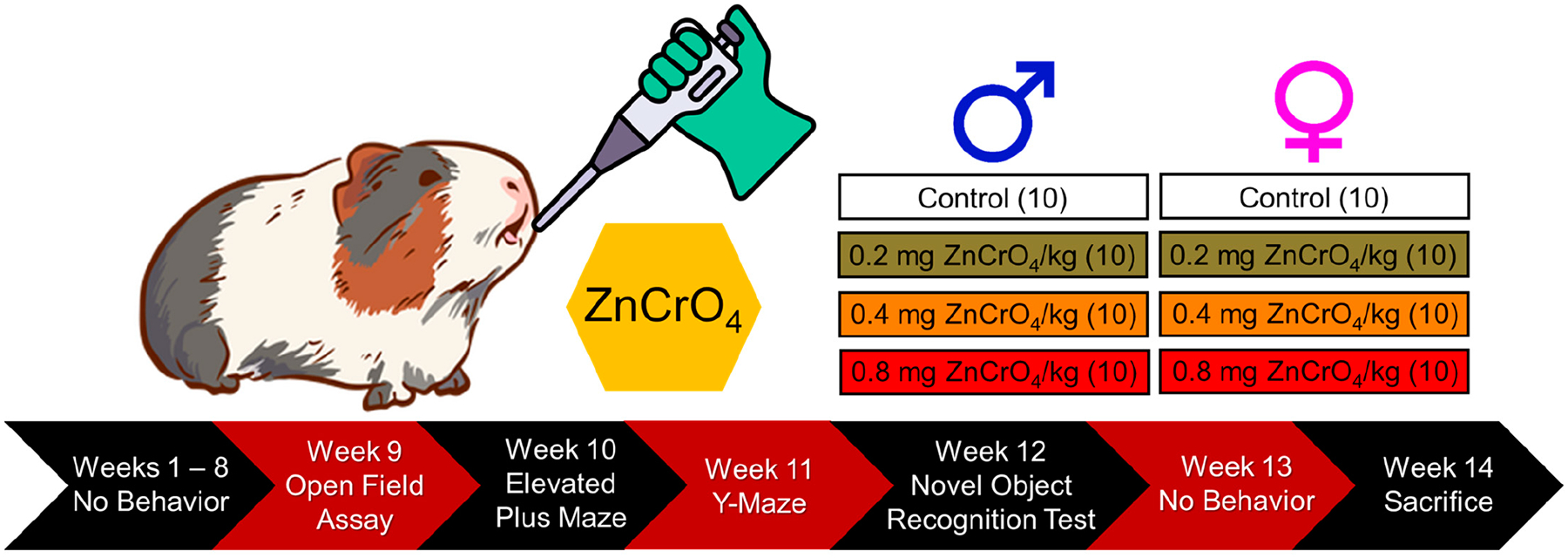
Study design to assess the toxic effects of zinc chromate (ZnCrO_4_) on guinea pig behavior and health after 90 days of exposure via oropharyngeal aspiration. Some animals were lost to attrition during exposure, with death not necessarily related to Cr(VI) exposure. Animals lost to attrition include: 1 control male, 1 male exposed to 0.2 mg/kg, 1 male exposed to 0.4 mg/kg, 1 male exposed to 0.8 mg/kg, and 1 female exposed to 0.4 mg/kg.

Guinea pigs were divided into four study groups, with each group containing 10 animals: control, 0.2, 0.4, and 0.8 mg/kg. Guinea pigs were exposed to zinc chromate once per week via oropharyngeal aspiration for 90 days, beginning the exposures at 70–76 days old. Study design is shown in Figure 1. We previously reported that this exposure regimen in rats resulted in lung Cr levels that were consistent with studies monitoring lung Cr levels in chromate workers [28]. Guinea pigs were exposed at the same time of day, on the same day of the week, each week, for the duration of the study. Zinc chromate (Z00277, Pfaltz and Bauer, Waterbury, CT, USA) was washed twice with distilled water and acetone to remove water-soluble compounds and organic contaminants. Once dry, zinc chromate particles were suspended in cold, filtered, double-distilled water and stirred overnight at 4 °C with a magnetic stir bar to create a stock solution of homogenized particle size. This method results in particle sizes ranging from 0.2 to 2.3 μm, with a mean size of 1.7 μm [29]. Zinc chromate dilutions were prepared in double-distilled water, and control animals were given 0.9% normal saline (Z1377, Cytiva, Wilmington, DE, USA).

Guinea pig body mass was recorded immediately prior to exposure each week, and the volume of zinc chromate administered to each guinea pig was calculated from this mass. The volume administered did not exceed 50 μL. For oropharyngeal aspiration exposures, guinea pigs were placed in an induction chamber (Kent Scientific Corporation, Torrington, CT, USA) and anesthetized with 4% isoflurane (502017, Vet One, MWI Animal Health, Boise, ID, USA) for approximately 4–5 min. Once anesthetized, guinea pigs were removed from the induction chamber and held in a supine position. The animal’s nostrils were blocked, the mouth was held open, and the tongue was gently pulled aside with forceps such that it did not block the airway. Using a micropipette, the calculated volume of zinc chromate was administered at the back of the throat. Nostrils were held pinched until the guinea pig took 15 deep breaths or exhibited a cough reflex. Guinea pigs were then returned to their home cage and monitored until they resumed normal activity. Guinea pigs in the control groups underwent the same exposure parameters, but were administered a saline solution instead. Some animals died unexpectedly during weeks 1–8, with death not necessarily related to Cr(VI) exposure, including 1 control male, 1 male exposed to 0.2 mg/kg, 1 male exposed to 0.4 mg/kg, 1 male exposed to 0.8 mg/kg, and 1 female exposed to 0.4 mg/kg. However, tissues were collected from most of these animals for metals analysis.

After 90 days, guinea pigs were anesthetized via intraperitoneal injection of 120 mg/kg of 1:12 ketamine:xylazine. Guinea pigs were euthanized via exsanguination, using cardiac perfusion of filtered, cold, 1x DPBS without calcium or magnesium (20–031-CV, Corning, Glendale, AZ, USA). Guinea pigs were then decapitated for brain tissue collection. Brains were removed, rinsed in cold 1x DPBS, blotted to remove excess liquid, and bisected using a guinea pig brain matrix (Visikol, Hampton, NJ). Brains were micro-dissected to isolate the hippocampus for metallomic analyses. Samples were placed in 15 mL trace element-free tubes and stored at −80 °C until digestion.

### 2.3. Behavior Analyses

Behavior chambers were constructed by Dr. Jun Cai (Pediatrics Research Institute, University of Louisville), as previously described [21,22]. All behavior assays were performed under red light. All chambers were placed inside a space enclosed by four poster-board walls to limit exposure to ambient light and noise. Guinea pigs performed behavior assays one day after exposure during the 9th, 10th, 11th, and 12th weeks of the study (e.g., guinea pigs exposed on Monday performed behavior assays on Tuesday). Table 1 describes the schedule of behavior assays. Guinea pigs performed each behavior assay in the afternoon to limit the influence of circadian rhythm. Cages were covered during transport to limit anxiety.

We recorded aerial views of each experiment using a 4 mm C Series Fixed Focal Length Lens camera (33300, Edmund Optics, Barrington, NJ, USA). All behavior experiments were recorded and analyzed with the ANY-maze Video Tracking System (v.7.33, Stoelting Co., Wood Dale, IL, USA). All chambers and mazes were cleaned with 70% ethanol between trials.

### 2.4. Open Field Assay

Guinea pigs performed the open field assay during the 9th week of exposure. The open field chamber consisted of a square, open area surrounded by four walls. One wall was made of plexiglass to enable recording from the side. Dimensions of the open field assay were 71 cm × 71 cm × 43 cm (L × W × H). Guinea pigs were placed in the center of the chamber and allowed to freely explore the maze for 10 min. In the open field assay, we assessed anxiety and fear-related behaviors (center area exploration, freezing behaviors), as well as locomotor function (distance traveled, maximum speed attained) [30,31]. The perimeter of the center area was defined as 14.2 cm from the walls, twice the width of a guinea pig. Guinea pigs were determined to be in the center area if their entire body mass was within this perimeter. Freezing describes extended periods of immobility. We defined a threshold for freezing as 10 s, meaning an animal was considered frozen if it remained immobile for more than 10 s. Some guinea pigs exhibited limited participation in the open field assay. To account for skewed data from “non-participants,” we assessed the distance traveled by each sex, independent of exposure group, and removed guinea pigs in the bottom 10th percentile from consideration. We removed males who traveled fewer than 0.4 m (4 guinea pigs) and females who traveled fewer than 2.5 m (3 guinea pigs). Guinea pigs statistically removed from open field assay analysis include 2 control males, 1 male exposed to 0.4 mg/kg, 1 male exposed to 0.8 mg/kg, 1 control female, and 2 females exposed to 0.4 mg/kg.

### 2.5. Elevated Plus Maze

Guinea pigs performed the elevated plus maze during the 10th week of exposure. The elevated plus maze consisted of a platform with four arms in the shape of a “+,” and elevated 74.5 cm from the floor. Two arms of the maze are open (without walls), and two arms are enclosed on three sides by walls. Each arm was 51 cm × 15 cm, and the walls around the enclosed arms were 43 cm tall. The four arms of the maze were connected by a central 15 cm × 15 cm square. Guinea pigs were placed in the elevated plus maze, facing an open arm, and allowed to freely explore the maze for 5 min. We assessed time spent exploring each area of the maze (open arm, closed arm, center area) as measures of anxiety [32,33]. Arm entry was defined as 80% of a guinea pig’s mass being in a single arm. A guinea pig was defined as being in the center area of the elevated plus maze if less than 75% of the animal was in any of the four arms. One guinea pig (0.8 mg/kg, female) was removed from analyses due to two falls from the elevated platform.

### 2.6. Y-Maze

Guinea pigs performed the Y-maze during the 11th week of exposure. The Y-maze consisted of three radial arms around a central triangle forming the shape of a “Y,” fully enclosed by walls. Each arm of the maze was 51 cm × 15 cm × 43 cm (L × W × H), and the arms were connected by an equilateral triangle with 15 cm sides. Guinea pigs were placed in the center and allowed to freely explore the maze for 8 min. We assessed spatial memory as changes in non-alternating arm entry [34]. Non-alternating arm entry occurs when an animal re-enters the same arm one or more times in a sequence of three arm entries. Rodents prefer to explore novel areas; thus, an increase in the percentage of non-alternations performed indicates an impairment of spatial memory. Percent non-alternations was calculated using the following equation:

$$\frac{\left(\text{Number of NonAlternations}\right)}{\left(\text{Number of Alternations}+\text{Number of NonAlternations}\right)}\times 100$$

Entry into each arm was defined as at least 80% of the guinea pig’s mass being in a single arm. Exit from an arm was defined as less than 20% of the guinea pig’s mass remaining in a single arm. If guinea pigs performed fewer than 5 arm entries, they were deemed “non-participants” and excluded from analyses. Guinea pigs performing 5 or fewer arm entries completed a maximum of 3 sequences, which heavily skews the mean percentage of non-alternations performed by the group. We removed any guinea pigs completing fewer than 5 arm entries (17 guinea pigs) to better assess Cr(VI) effects in animals who actively participated in the assay. Guinea pigs removed from analysis include, 3 control males, 2 males exposed to 0.2 mg/kg, 3 males exposed to 0.4 mg/kg, 4 males exposed to 0.8 mg/kg, 1 control female, 2 females exposed to 0.2 mg/kg, 2 females exposed to 0.4 mg/kg, and 1 female exposed to 0.8 mg/kg.

### 2.7. Novel Object Recognition Test

Guinea pigs performed the novel object recognition test during the 12th week of exposure. The novel object recognition test was performed in the same chamber as the open field assay. The test was completed in two 5 min trials, 25 min apart. During trial 1, two 3.8 cm^3^ wood blocks were placed in opposite corners of the chamber, along the wall made of plexiglass. The wood blocks were identical to enrichment objects given to guinea pigs in their home cages and served as “familiar objects.” Blocks were placed 7 cm from both walls, allowing space for the guinea pig to explore the chamber around the objects without interacting with them. Guinea pigs were placed in the center of the chamber with the familiar objects and allowed to freely explore for 5 min. Guinea pigs were then removed from the chamber and returned to their cage for 25 min. During trial 2, one of the wood blocks was replaced with a 4.8 cm × 4.9 cm × 5.2 cm (L × W × H) novel object; the novel object was a cylindrical, anthropomorphized clay statuette. Texture, shape, color, and size of the novel object are drastically distinct from those of the familiar objects. Familiar and novel objects were adhered to the bottom of the chamber with Velcro to prevent the guinea pigs from moving the objects. The positions of the objects were alternated between animals to limit preference for a corner of the chamber. We assessed time spent interacting with the familiar or novel object in trial 2 as a measure of working memory, which we present as percent preference. Percent preference was calculated using the following equation:

$$\frac{\left(\text{Time Exploring Novel Object}\right)}{\left(\text{Time Exploring Familiar Object}+\text{Time Exploring Novel Object}\right)}\times 100$$

Changes in exploration during the novel object recognition test are associated with working memory impairments or a preference for familiar objects [35]. Some guinea pigs exhibited limited participation in this assay. We again removed any guinea pigs who fell in the bottom 10th percentile for total object exploration (time spent around familiar and novel objects combined) during trial 2 of the novel object recognition test (less than 12 s). We identified four guinea pigs as “non-participants” and removed all four from analyses. Guinea pigs removed from analysis include: 1 control male, 1 male exposed to 0.2 mg/kg, 2 males exposed to 0.4 mg/kg, 2 males exposed to 0.8 mg/kg, and 2 females exposed to 0.4 mg/kg.

### 2.8. Metallomics Analyses

We assessed metallomics with inductively coupled plasma-mass spectrometry (ICPMS) in the Integrative Molecular Analysis Core (IMAC) at the University of New Mexico. For ICP-MS analyses, samples were prepared as previously described [20]. The hippocampus was selected for analysis based on our previous results indicating Cr selectively accumulated in this brain region. Tissues were digested in 70% nitric acid at 85 °C for 3 h. After digestion, tissues were incubated with 100 μL 3% hydrogen peroxide for 1 h at room temperature, before being diluted to a final concentration of 5% nitric acid and filtered through Acrodisc 32 mm 0.45 μm Supor^®^ filters (4654, Pall Corporation, Washington, NY, USA) into trace element-free 15 mL centrifuge tubes (89049–170, VWR Avantor, Radnor, PA, USA). Filtered digestates were stored at −20 °C until ICP-MS analyses. Samples were run alongside a reference standard for trace elements in water (NIST1643F, Millipore Sigma, Burlington, MA, USA). Samples were analyzed on an Agilent 7900 ICP-MS. Initial calibration blank and initial calibration verification standards were run at the start of the analysis, after every 10 samples, and at the end of the analysis. An internal standard (5188–6525, Agilent, Santa Clara, CA, USA) was included in all sample runs to account for any matrix effects; it contains lithium (Li), scandium (Sc), germanium (Ge), rhodium (Rh), indium (In), terbium (Tb), and bismuth (Bi). Any samples below the detection limit were reported as ½ the limit of detection. For reporting results of metal levels, we limited the use of numbers after the decimal to metals with mean values less than 1000 ng/g. The final sample sizes for study groups were 9, 9, 9, and 10 for control, 0.2, 0.4, and 0.8 mg/kg male study groups, and 10 animals per group for all female groups. We considered these data with and without the animals that died early, and observed no statistical impact on the results reported below.

### 2.9. Statistical Analyses

Statistical analyses were conducted using GraphPad Prism 9 (v.9.5.1). We assessed normality using the Anderson–Darling test (α = 0.05). Based on the results of the normality test, we assessed statistical significance with a Student’s *t*-test or Mann–Whitney U Test for parametric and non-parametric data, respectively. Statistical significance was tested for all groups across all assays, and reported where *p* < 0.05, though comparisons where *p* < 0.1 are noted. Data are expressed as mean ± SEM.

## 3. Results

For all endpoints assessed, we first report Cr(VI) effects with both sexes pooled, before comparing Cr(VI) effects across sexes.

### 3.1. Cr(VI) Altered Behaviors in the Open Field Assay

**Figure 2.**
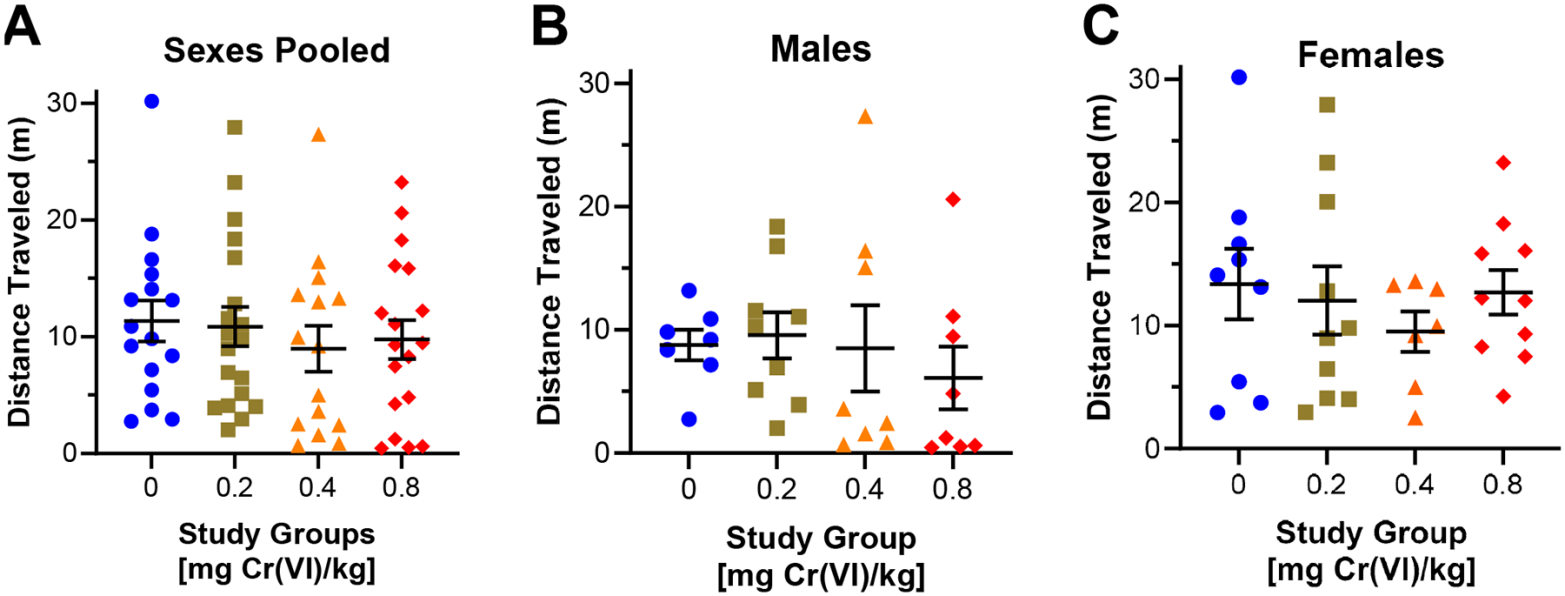
Cr(VI) had a weak effect on distance traveled in the open field assay. We assessed the effects of Cr(VI) on distance traveled in the open field assay after 9 weeks of exposure. (**A**) Distance traveled in both sexes combined, we observed a decrease in guinea pigs exposed to 0.4 mg/kg. N = 16, 19, 15, and 18 for control, 0.2 mg/kg, 0.4 mg/kg, and 0.8 mg/kg study groups, respectively. (**B**) Distance traveled in males exhibited a decrease after 0.8 mg/kg. N = 7, 9, 8, and 8 for control, 0.2 mg/kg, 0.4 mg/kg, and 0.8 mg/kg study groups, respectively. (**C**) Distance traveled in females only exhibited a decrease in guinea pigs exposed to 0.4 mg/kg. N = 9, 10, 7, and 10 for controls, 0.2 mg/kg, 0.4 mg/kg, and 0.8 mg/kg study groups, respectively. Blue circles are control groups, gold squares are 0.2 mg/kg, orange triangles are 0.4 mg/kg, and red diamonds are 0.8 mg/kg. Bars represent mean ± SEM. Normality was assessed using an Anderson–Darling Test. Statistical significance was determined using a *t*-test with Welch’s Correction (parametric data) or a Mann–Whitney Test (non-parametric data).

**Figure 3.**
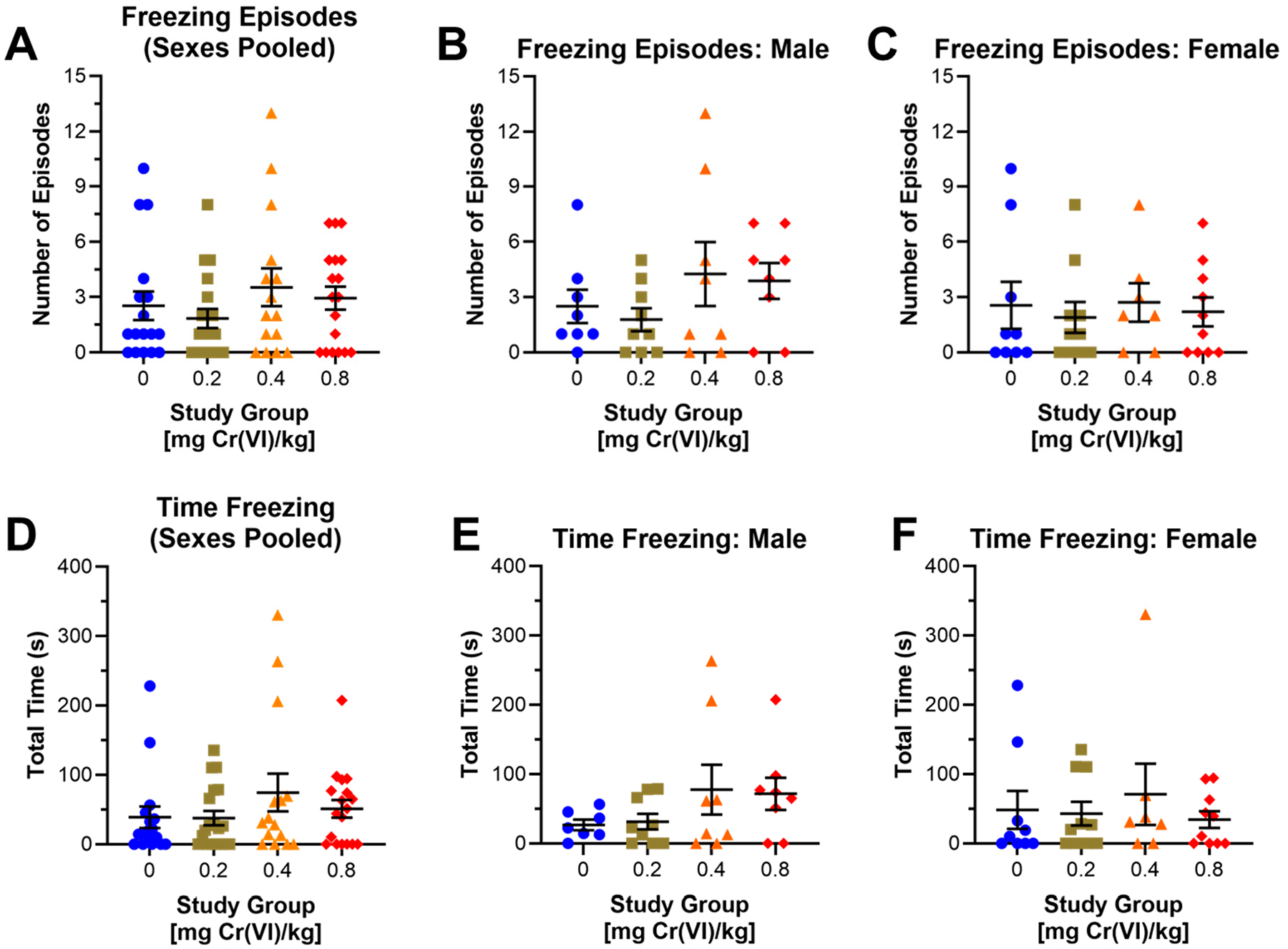
Cr(VI) had little effect on freezing behavior in guinea pigs. We assessed freezing behavior in the open field assay after 9 weeks of Cr(VI) exposure. (**A**,**D**) Cr(VI) had little effect on freezing episodes across exposure groups when sexes were pooled, and non-significantly increased time spent freezing in 0.4 mg/kg exposed guinea pigs. N = 16, 19, 15, and 18 for control, 0.2 mg/kg, 0.4 mg/kg, and 0.8 mg/kg study groups, respectively. (**B**,**E**) Similarly, Cr(VI) did not affect freezing episodes in exposed males, but increased freezing time in groups exposed to 0.4 and 0.8 mg/kg by 50.79 and 44.82 s, respectively. N = 7, 9, 8, and 8 for control, 0.2 mg/kg, 0.4 mg/kg, and 0.8 mg/kg study groups, respectively. (**C**,**F**) No apparent effects in Cr(VI)-exposed female groups on freezing episodes or time. N = 9, 10, 7, and 10 for control, 0.2 mg/kg, 0.4 mg/kg, and 0.8 mg/kg study groups, respectively. Blue circles are control groups, gold squares are 0.2 mg/kg, orange triangles are 0.4 mg/kg, and red diamonds are 0.8 mg/kg. Bars represent mean ± SEM. Normality was assessed using an Anderson–Darling Test. Statistical significance was determined using a *t*-test with Welch’s Correction (parametric data) or a Mann–Whitney Test (non-parametric data).

**Figure 4.**
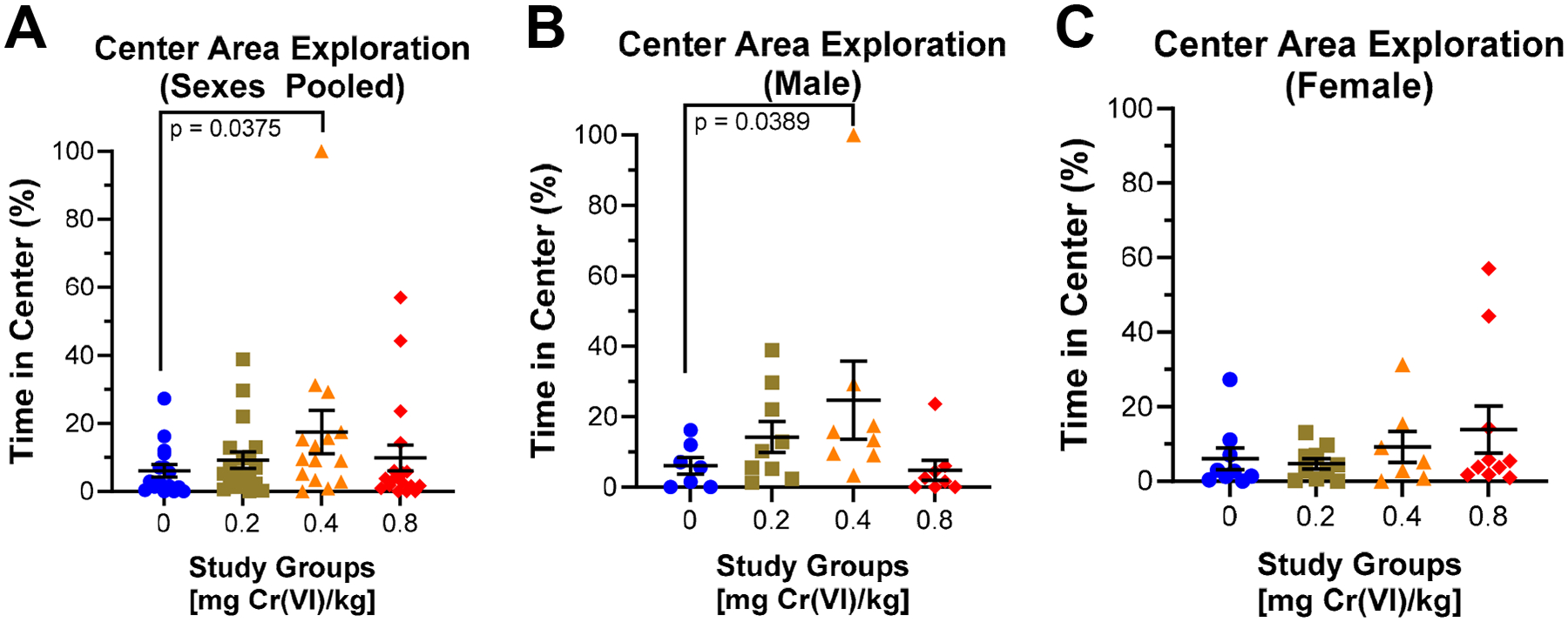
Cr(VI) increased center exploration during the open field assay. We assessed the effects of Cr(VI) on time spent in the center area during the open field assay after 9 weeks of exposure. (**A**) Considering the sexes pooled, we observed an increase in guinea pigs exposed to 0.4 mg/kg. N = 16, 19, 15, and 18 for control, 0.2 mg/kg, 0.4 mg/kg, and 0.8 mg/kg study groups, respectively. (**B**) Males exhibited a concentration-associated increase after exposure to 0.2 or 0.4 mg/kg. N = 7, 9, 8, and 8 for control, 0.2 mg/kg, 0.4 mg/kg, and 0.8 mg/kg study groups. (**C**) Females exhibited a 7.85% increase in guinea pigs exposed to 0.8 mg/kg. N = 9, 10, 7, and 10 for control, 0.2 mg/kg, 0.4 mg/kg, and 0.8 mg/kg study groups, respectively. Blue circles are control groups, gold squares are 0.2 mg/kg, orange triangles are 0.4 mg/kg, and red diamonds are 0.8 mg/kg. Bars represent mean ± SEM. Normality was assessed using an Anderson–Darling Test. Statistical significance was determined using a *t*-test with Welch’s Correction (parametric data) or a Mann–Whitney Test (non-parametric data).

**Figure 5.**
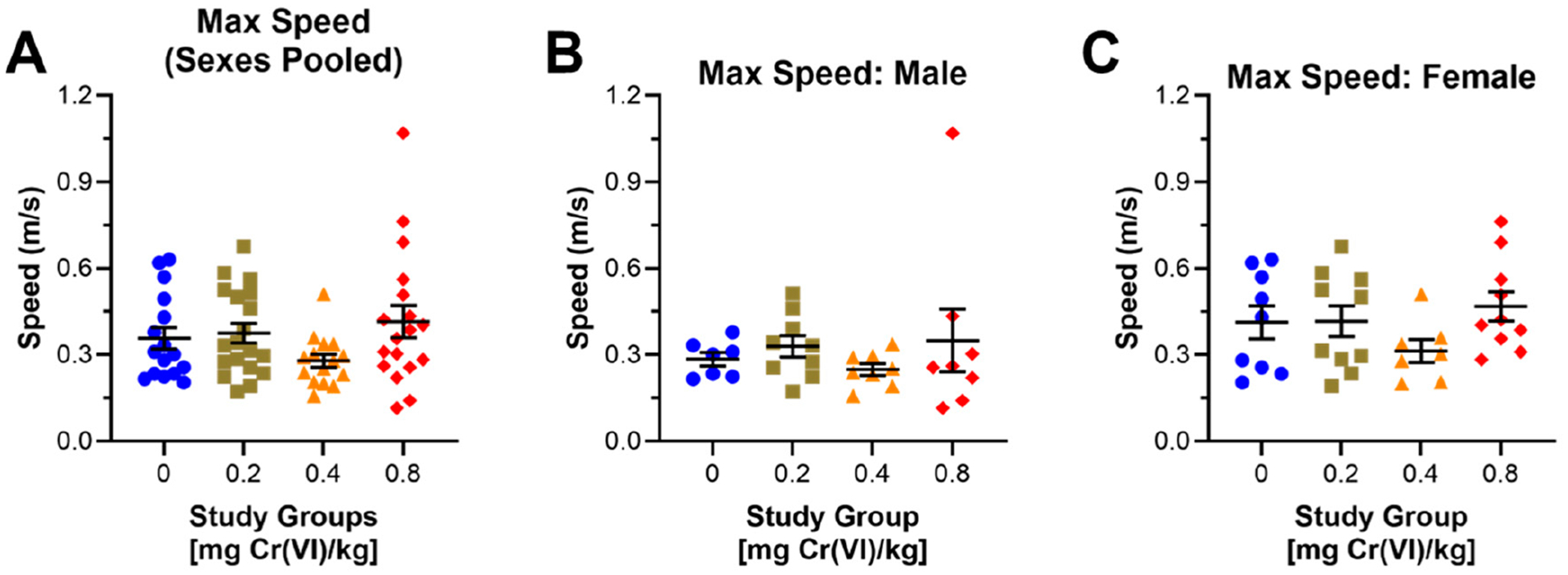
Cr(VI) increased the maximum speed of males during the open field assay. We assessed maximum speed in the open field assay during the 9th week of exposure. (**A**) Pooling sexes, Cr(VI) increased maximum speed by 0.06 m/s in 0.8 mg/kg exposed animals but decreased maximum speed by 0.08 m/s in 0.4 mg/kg exposed animals. N = 16, 19, 15, and 18 for control, 0.2 mg/kg, 0.4 mg/kg, and 0.8 mg/kg study groups, respectively. (**B**) Cr(VI) increased maximum speed by 0.04 and 0.03 m/s in 0.2 and 0.8 mg/kg exposed males. N = 7, 9, 8, and 9 for control, 0.2 mg/kg, 0.4 mg/kg, and 0.8 mg/kg study groups, respectively. (**C**) Females exhibited a 1.0 m/s maximum speed after exposure to 0.4 mg/kg, but a 0.06 m/s increase in maximum speed after exposure to 0.8 mg/kg. N = 9, 10, 7, and 10 for control, 0.2 mg/kg, 0.4 mg/kg, and 0.8 mg/kg study groups, respectively. Blue circles are control groups, gold squares are 0.2 mg/kg, orange triangles are 0.4 mg/kg, and red diamonds are 0.8 mg/kg. Bars represent mean ± SEM. Normality was assessed using an Anderson–Darling Test. Statistical significance was determined using a *t*-test with Welch’s Correction (parametric data) or a Mann–Whitney Test (non-parametric data).

Guinea pigs performed the open field assay during the 9th week of exposure. In the open field assay, we measured distance traveled (Figure 2), freezing behavior (Figure 3), center area exploration (Figure 4), and maximum speed attained (Figure 5).

We assessed distance traveled in the open field assay as a measure of locomotor performance. Considering our data with sexes pooled, we observed a 2.4 m decrease in the distance traveled by guinea pigs exposed to 0.4 mg/kg (Figure 2A), with mean distances of 11.4, 10.9, 9.0, and 9.8 m for control, 0.2, 0.4, and 0.8 mg/kg, respectively. Considering sexes separately, males exhibited a nonsignificant decrease by 1.37 m in distance traveled after exposure to 0.8 mg/kg, though there is wide variability within groups (Figure 2B); while females exhibit a nonsignificant decrease by 3.1 m after exposure to 0.4 mg/kg (Figure 2C)—males, 8.8, 9.6, 8.5, 6.1 m for control, 0.2, 0.4, and 0.8 mg/kg, respectively, females, 13.4, 12.0, 9.5, and 12.7 m for control, 0.2, 0.4, and 0.8 mg/kg, respectively.

We assessed freezing behavior in the open field assay to evaluate anxiety or fear, measured as the number of freezing episodes and the total time freezing. Cr(VI) induces a nonsignificant increase in freezing episodes (1.0 episodes) and total time freezing (35.5 s) in 0.4 mg/kg exposed animals when sexes are pooled (Figure 3A,D): 39.0, 37.8, 74.6, and 51.0 s for control, 0.2, 0.4, and 0.8 mg/kg, respectively. Comparing across sexes, effects were more noticeable in males than in females; however, we did not observe statistically significant changes. In males, Cr(VI) induced a nonsignificant increase in freezing episodes by males exposed to 0.4 mg/kg (1.75 episode increase). Males exposed to 0.4 and 0.8 mg/kg spent more time frozen compared to controls (50.8 and 44.8 s longer, respectively). (Figure 3B,E): 26.9, 31.6, 77.7, and 71.7 s for control, 0.2, 0.4, and 0.8 mg/kg, respectively. Cr(VI)-exposed females exhibit little to no change in the number of freezing episodes or the time freezing relative to controls (Figure 3C,F).

We assess center exploration as the percentage time spent in the center area (Figure 4). Center exploration is significantly increased in guinea pigs exposed to 0.4 mg/kg, when comparing across pooled sexes (Figure 4A): 6.1%, 9.2%, 17.5%, and 9.8% for control, 0.2, 0.4, and 0.8 mg/kg, respectively. In males, center area exploration is significantly increased in guinea pigs exposed to 0.4 mg/kg (Figure 4B): 6.1%, 14.2%, 24.7%, and 4.8% for control, 0.2, 0.4, and 0.8 mg/kg, respectively. Cr(VI) increased center exploration by 7.85% in females exposed to 0.8 mg/kg, though this effect is not significant (Figure 4C).

We assessed the maximum speed of guinea pigs during the open field assay exploration (Figure 5). Pooling sexes, maximum speed was faster by 0.06 m/s in 0.8 mg/kg exposed groups, but 0.08 m/s slower in 0.4 mg/kg exposed groups (Figure 5A): 0.32, 0.38, 0.26, and 0.40 m/s for control, 0.2, 0.4, and 0.8 mg/kg, respectively. 0.36, 0.38, 0.28, and 0.42 m/s for control, 0.2, 0.4, and 0.8 mg/kg, respectively. Maximum speed is 0.06 m/s faster in males after exposure to 0.8 mg/kg, but we observed no effect in other exposure groups (Figure 5B): 0.29, 0.33, 0.25, and 0.35 m/s for control, 0.2, 0.4, and 0.8 mg/kg, respectively. Cr(VI) exposure decreased maximum speed in females exposed to 0.4 mg/kg by 0.10 m/s, but maximum speed in females exposed to 0.8 mg/kg was 0.06 m/s faster (Figure 5C): 0.41, 0.42, 0.31, and 0.47 m/s for control, 0.2, 0.4, and 0.8 mg/kg, respectively.

### 3.2. Cr(VI) Altered Anxiety in the Elevated Plus Maze

**Figure 6.**
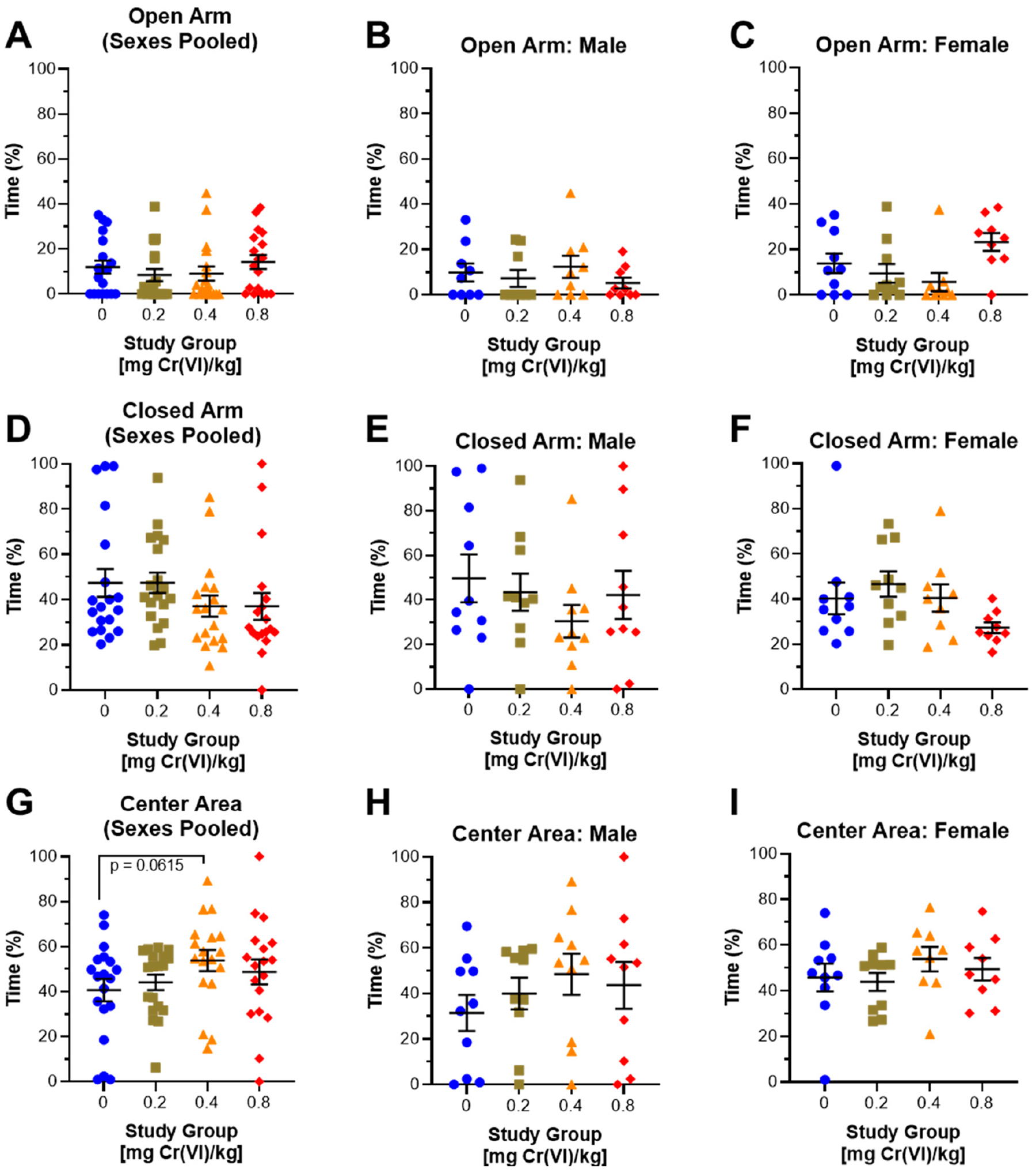
Cr(VI) altered anxiety-like behavior in the elevated plus maze. We assessed time spent exploring the open arms, closed arms, and center area of the elevated plus maze after 10 weeks of exposure. When we pooled sexes, we observed 3.55% and 2.97% decreases in on open arm exploration by 0.2 and 0.4 mg/kg exposed animals, but a 2.27% increase in open arm exploration by 0.8 mg/kg exposed animals (**A**). We observed a 10.2% and 10.4% decrease in closed arm exploration (**D**) and a 13.2% and 8.2% increase in center area exploration by 0.4 and 0.8 mg/kg exposed animals (**G**); N = 19, 19, 18, and 18 for control, 0.2 mg/kg, 0.4 mg/kg, and 0.8 mg/kg study groups, respectively. Males exhibited a 2.62 and 4.66% decrease in open arm exploration by 0.2 and 0.8 mg/kg exposed animals, but a 2.51% increase in 0.8 mg/kg exposed animals (**B**). We observed decreased closed arm exploration in males (6.2%, 19.5%, and 7.5% for 0.2, 0.4, and 0.8 mg/kg exposed animals, respectively), (**E**) and a corresponding increase in center area exploration by all exposed males (**H**); N = 9 for all groups. We observed decreased open arm exploration by females exposed to 0.2 or 0.4 mg/kg, but we observed an increase in females exposed to 0.8 mg/kg (**C**); conversely, we observed increased closed arm exploration by females, but a decrease in those exposed to 0.8 mg/kg (**F**); we observed no effect on center area exploration by females (**I**); N = 10, 10, 9, and 9 for control, 0.2 mg/kg, 0.4 mg/kg, and 0.8 mg/g study groups, respectively. Blue circles are control groups, gold squares are 0.2 mg/kg, orange triangles are 0.4 mg/kg, and red diamonds are 0.8 mg/kg. Bars represent mean ± SEM. Normality was assessed using an Anderson–Darling Test. Statistical significance was determined using a *t*-test with Welch’s Correction (parametric data) or a Mann–Whitney Test (non-parametric data).

Guinea pigs performed the elevated plus maze during the 10th week of exposure. We assessed anxiety by measuring the time spent exploring the open arms, closed arms, and center area. Cr(VI) had decreased open arm exploration by 3.55% and 2.97% in 0.2 and 0.4 mg/kg exposed groups but increased open arm exploration by 2.27% in 0.8 mg/kg exposed groups when sexes were pooled; however, we observed a 10.2% and 10.4% decrease in closed arm exploration, alongside a 13.2% and 8.2% increase in center area exploration, by guinea pigs exposed to 0.4 and 0.8 mg/kg, respectively, (Figure 6A,D,G): 47.4%, 47.5%, 37.2%, and 37.0% closed arm exploration for control, 0.2, 0.4, and 0.8 mg/kg, respectively, 40.6%, 44.1%, 53.8%, and 48.8% time in center for control, 0.2, 0.4, and 0.8 mg/kg, respectively.

Considering sexes separately, males exhibited decreased open arm exploration by 2.62% and 4.66% in 0.2 and 0.8 mg/kg exposed animals, but males exhibited a 2.51% increase following exposure to 0.8 mg/kg (Figure 6B). We observed 6.2%, 19.5%, and 7.5% decreased time exploring the closed arms and 8.6%, 17%, and 12.2% increased time in the center area for 0.2, 0.4, and 0.8 mg/kg exposed animals, respectively, (Figure 6B,E,H)—49.7%, 43.5%, 30.4%, and 42.2% closed arm exploration, 31.4%, 40.0%, 48.4%, and 43.6% time in center for control, 0.2, 0.4, and 0.8 mg/kg, respectively. In females, we observed 4.4% and 8.2% decreased open arm exploration after exposure to 0.2 and 0.4 mg/kg but a 9.4% increase in open arm exploration after exposure to 0.8 mg/kg (Figure 6C): 13.9%, 9.5%, 5.7%, and 23.3% open arm exploration for control, 0.2, 0.4, and 0.8 mg/kg, respectively. Females exposed to 0.2 mg/kg exhibited a 6.4% increase in closed arm exploration, but we observed a decrease (12.9%) in females exposed to 0.8 mg/kg (Figure 6F): 40.3%, 46.7%, 40.5%, and 27.4% for control, 0.2, 0.4, and 0.8 mg/kg, respectively. Cr(VI) did not affect female center area exploration (Figure 6I).

### 3.3. Cr(VI) Increased Y-Maze Non-Alternations in Males

**Figure 7.**
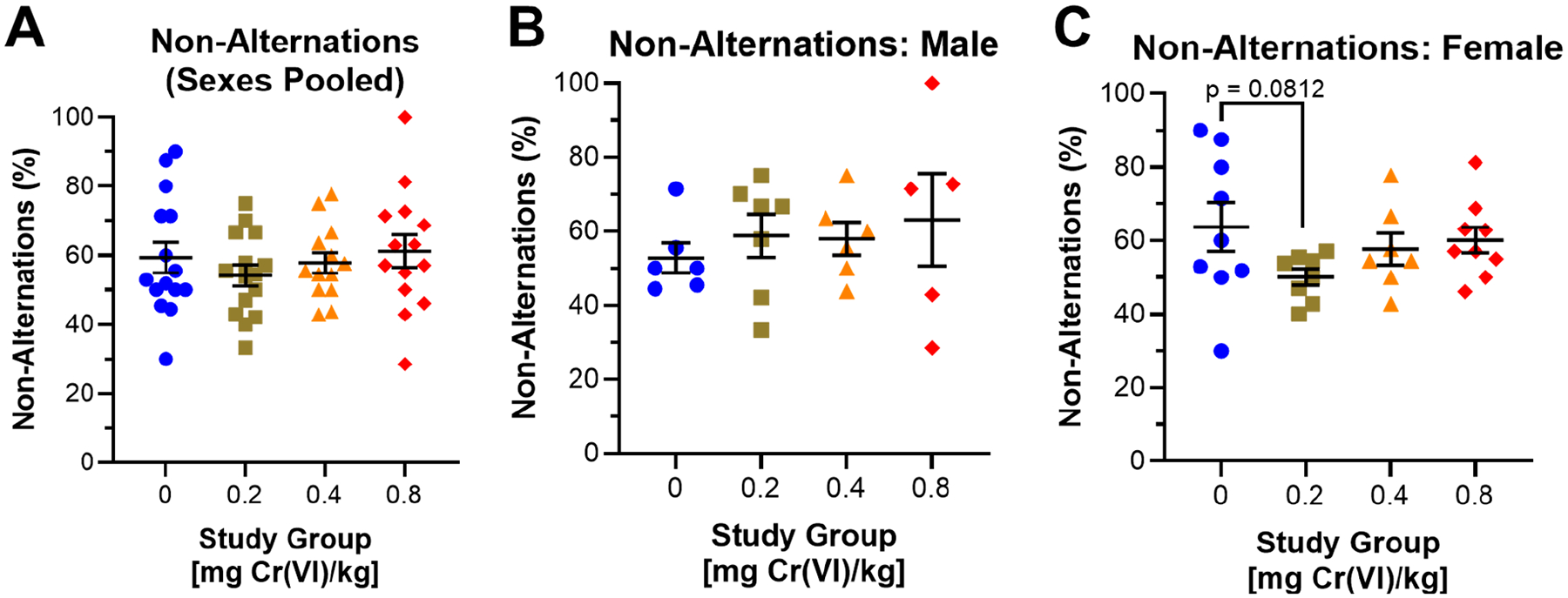
Cr(VI) impacted non-alternations during the Y-maze. We assessed the percentage of spontaneous non-alternations in the Y-Maze after 11 weeks of exposure. (**A**) Cr(VI) did not affect the percent spontaneous non-alternations when considering sexes pooled. N = 15, 15, 13, and 14 for control, 0.2 mg/kg, 0.4 mg/kg, and 0.8 mg/kg study groups, respectively. (**B**) Percent spontaneous non-alternations was increased by 6%, 5.2%, and 10.3% in 0.2, 04, and 0.8 mg/kg exposed males, respectively. N = 6, 7, 6, and 5 for control, 0.2 mg/kg, 0.4 mg/kg, and 0.8 mg/kg study groups, respectively. (**C**) Cr(VI) decreased the percentage of spontaneous non-alternations in females exposed to 0.2 mg/kg but had no effect in other groups. N = 9, 8, 7, and 9 for control, 0.2 mg/kg, 0.4 mg/kg, and 0.8 mg/kg study groups, respectively. Blue circles are control groups, gold squares are 0.2 mg/kg, orange triangles are 0.4 mg/kg, and red diamonds are 0.8 mg/kg. Bars represent mean ± SEM. Normality was assessed using an Anderson–Darling Test. Statistical significance was determined using a *t*-test with Welch’s Correction (parametric data) or a Mann–Whitney Test (non-parametric data).

Guinea pigs performed the Y-maze during the 11th week of exposure. We assessed the percent of spontaneous non-alternations as a measure of spatial memory. Considering the effects of Cr(VI) on spatial memory, regardless of sex, we observed no effect (Figure 7A). Considering sexes separately, we observed a 6%, 5.2%, and 10.3% increase in non-alternations for males exposed to 0.2, 0.4, and 0.8 mg/kg (Figure 7B): 52.8%, 58.8%, 58.0% and 63.1% for control, 0.2, 0.4, and 0.8 mg/kg, respectively. We observed decreased spontaneous non-alternations in females exposed to 0.2 mg/kg (13.7% decrease), but no effect in other groups (Figure 7C): 63.8%, 50.1%, 57.7%, and 60.2% for control, 0.2, 0.4, and 0.8 mg/kg, respectively.

### 3.4. Cr(VI) Increased Preference for Novel Objects in Females

**Figure 8.**
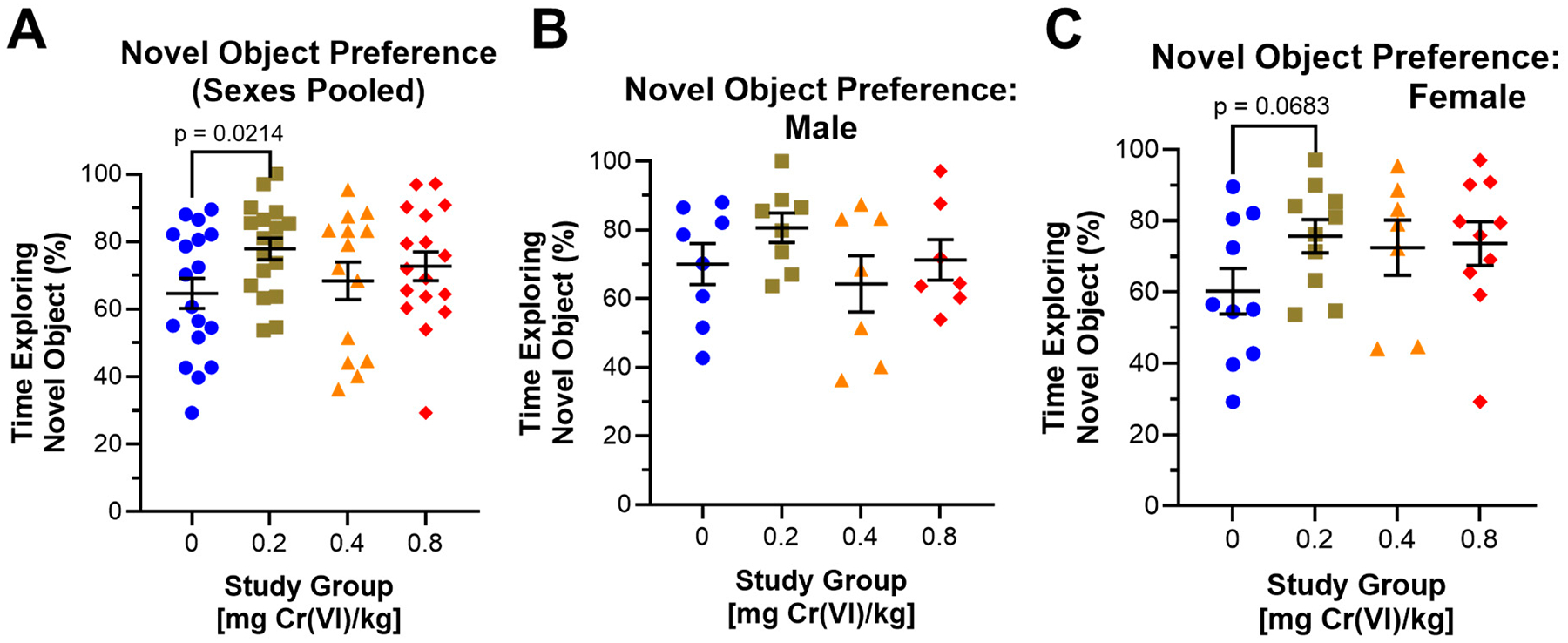
Cr(VI) exposure increased novelty preference. We assessed novel object preference in the novel object recognition test during the 12th week of exposure. (**A**) Considering sexes pooled, Cr(VI) exposure significantly increased novel object preference in 0.2 mg/kg exposed animals and increased novel object preference by 8.1% in 0.8 mg/kg exposed animals. N = 18, 18, 14, and 17 for control, 0.2 mg/kg, 0.4 mg/kg, and 0.8 mg/kg study groups, respectively. (**B**) Novel object preference was increased by 10.6% in males exposed to 0.2 mg/kg. N = 8, 8, 7, and 7 for control, 0.2 mg/kg, 0.4 mg/kg, and 0.8 mg/kg study groups, respectively. (**C**) Novel object preference was increased by 15.4%, 12.2%, and 13.3% increases for 0.2, 0.4, and 0.8 mg/kg exposed females, respectively. N = 10, 10, 7, and 10 for control, 0.2 mg/kg, 0.4 mg/kg, and 0.8 mg/kg study groups, respectively. Blue circles are control groups, gold squares are 0.2 mg/kg, orange triangles are 0.4 mg/kg, and red diamonds are 0.8 mg/kg. Bars represent mean ± SEM. Normality was assessed using an Anderson–Darling Test. Statistical significance was determined using a *t*-test with Welch’s Correction (parametric data) or a Mann–Whitney Test (non-parametric data).

Guinea pigs performed the novel object recognition test during the 12th week of exposure. We assessed the percent preference for the novel object in this assay as a measure of working memory and memory consolidation. Considering the effects of Cr(VI) on novel object preference, regardless of sex, we observed significantly increased novel object preference by guinea pigs exposed to 0.2 mg/kg (Figure 8A): 64.6%, 77.9%, 68.4%, and 72.7% for control, 0.2, 0.4, and 0.8 mg/kg, respectively. Considering sexes separately, males exposed to 0.2 mg/kg exhibited a 10.6% increase in preference for the novel object (Figure 8B): 70.1%, 80.7%, 64.3%, and 71.3% for control, 0.2, 0.4, and 0.8 mg/kg, respectively. In females, we observed increased preference for the novel object in all exposure groups (15.4%, 12.2%, and 13.3% increases for 0.2, 0.4, and 0.8 mg/kg exposed animals, respectively), (Figure 8C): 60.3%, 75.7%, 72.5%, and 73.6% for control, 0.2, 0.4, and 0.8 mg/kg, respectively.

### 3.5. Cr Accumulated in the Hippocampus and Induced Essential Metal Dyshomeostasis

**Figure 9.**
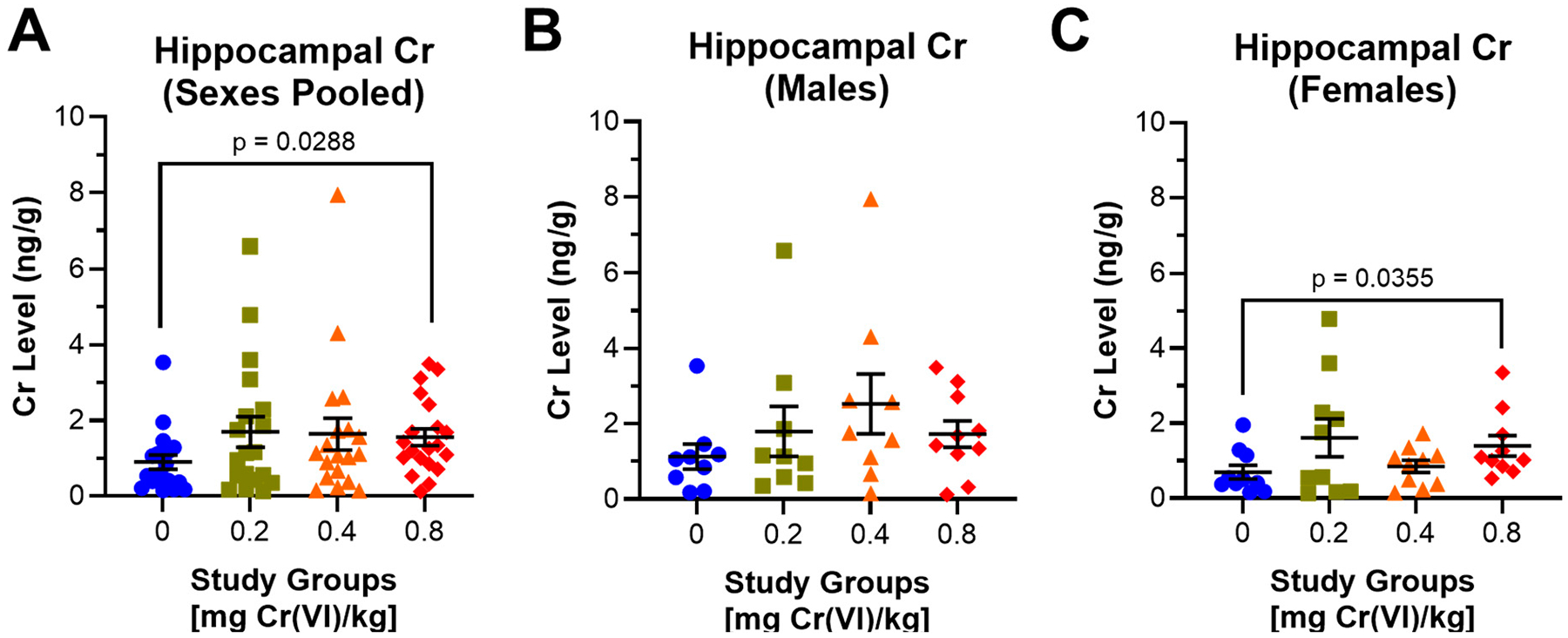
Hippocampal Cr increased in guinea pigs after 90 days of oropharyngeal aspiration. We measured hippocampal Cr accumulation by ICP-MS after 90 days of exposure to Cr(VI) by oropharyngeal aspiration. (**A**) After pooling data from both sexes, we observed significantly increased hippocampal Cr in guinea pigs exposed to 0.8 mg/kg. N = 19, 19, 19, and 20 for control, 0.2 mg/kg, 0.4 mg/kg, and 0.8 mg/kg study groups, respectively. (**B**) Males exhibited 0.67, 1.4, and 0.6 ng/g increases in hippocampal Cr for control, 0.2, 0.4, and 0.8 exposed males, respectively. N = 9, 9, 9, and 10 for control, 0.2 mg/kg, 0.4 mg/kg, and 0.8 mg/kg study groups, respectively. (**C**) Females exhibited significantly increased hippocampal Cr after exposure to 0.8 mg/kg. N = 10 for all groups. Blue circles are control groups, gold squares are 0.2 mg/kg, orange triangles are 0.4 mg/kg, and red diamonds are 0.8 mg/kg. Bars represent mean ± SEM. Normality was assessed using an Anderson–Darling Test. Statistical significance was determined using a *t*-test with Welch’s Correction (parametric data) or a Mann–Whitney Test (non-parametric data).

At the end of the study, we collected the hippocampus and assessed metallomics using ICP-MS. Considering these data regardless of sex, hippocampal Cr was elevated in all groups exposed to Cr(VI), with significantly higher Cr in guinea pigs exposed to 0.8 mg/kg (Figure 9A): 0.90, 1.70, 1.64, and 1.56 ng/g for control, 0.2, 0.4, and 0.8 mg/kg, respectively. Considering hippocampal Cr accumulation by sex, we observed increased hippocampal Cr in all male groups (0.67, 1.4, and 0.6 ng/g increases for 0.2, 0.4, and 0.8 mg/kg exposed animals, respectively), (Figure 9B): 1.13, 1.80, 2.53, and 1.73 ng/g for control, 0.2, 0.4, and 0.8 mg/kg, respectively. Females exhibited more pronounced Cr accumulation, with significantly elevated hippocampal Cr in females exposed to 0.8 mg/kg (Figure 9C): 0.69, 1.61, 0.85, and 1.39 for control, 0.2, 0.4, and 0.8 mg/kg, respectively.

**Figure 10.**
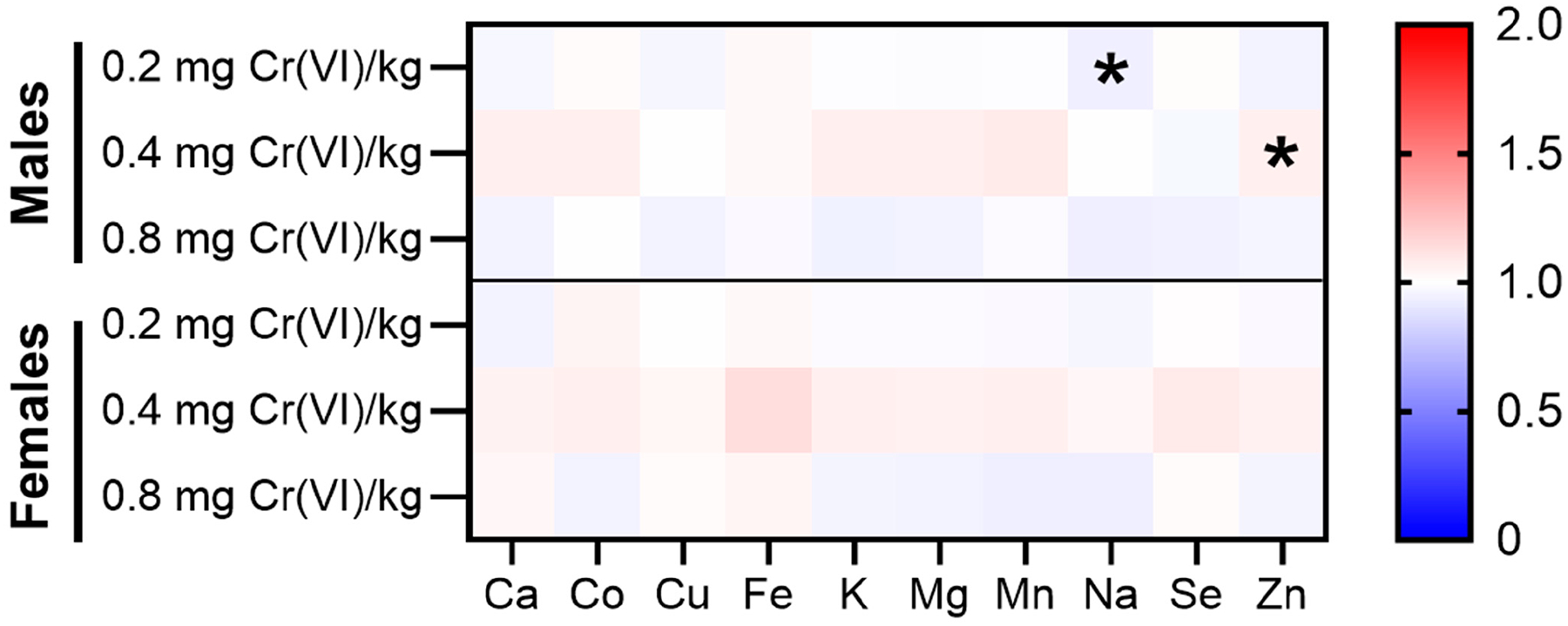
Exposure to Cr(VI) altered essential metal homeostasis in males, not in females. We assessed essential metals homeostasis in the hippocampus after 90 days of exposure to zinc chromate. Hippocampal essential metals were largely unaffected in females. Males exposed to 0.2 mg/kg exhibited a significant decrease in Na. Males exposed to 0.4 mg/kg exhibited a significant increase in Zn. For males, N = 9, 9, 9, and 10 for control, 0.2 mg/kg, 0.4 mg/kg, and 0.8 mg/kg study groups, respectively. For females, N = 10 for all groups. Normality was assessed using an Anderson–Darling Test. Statistical significance was determined using a *t*-test with Welch’s Correction (parametric data) or a Mann–Whitney Test (non-parametric data). Data represent mean fold-changes relative to sex-matched controls across groups. ZC = zinc chromate, * *p* < 0.05.

We assessed essential metal homeostasis in the hippocampus for the essential metals Ca, Co, Cu, Fe, K, Mg, Mn, Na, Se, and Zn. We report the fold-change in essential metals relative to sex-matched controls (Figure 10). Changes in female hippocampal essential metals were minimal, though it is worth noting that nearly all essential metals were increased in females exposed to 0.4 mg/kg. Males exhibited significant changes in homeostasis of Na and Zn, with decreased Na in males exposed to 0.2 mg/kg, while Zn was increased in males exposed to 0.4 mg/kg. Similarly to females, males exposed to 0.4 mg/kg exhibited increased levels for most metals. Raw values for male hippocampal essential metal levels are detailed in Table 2.

## 4. Discussion

This study demonstrated Cr(VI) neurotoxicity after 9–12 weeks of exposure to zinc chromate via oropharyngeal aspiration in guinea pigs, as indicated by impacts on behaviors and metallomics. To this point, few groups have addressed Cr(VI) neurotoxicity in occupationally relevant models, and most rodent Cr(VI) neurotoxicity studies used drinking water, oral gavage, or intraperitoneal injection to administer Cr(VI) [5,19–22,25,36]. One group demonstrated that exposure to welding fumes inhibited cortical function, but this group did not assess behaviors and used non-toxic Cr(III) as a representative compound [37]. Two studies exposed rats to Cr(VI) via intranasal instillation and reported behavioral effects, but anatomical differences in the nasal passages of rodents and humans present a challenge for translating this work between species, as rodents have a drastically more extensive nasal passage that reduces the distribution of particulates from the nose to the lungs or brain [11,12,38,39]. There is a key physiological distinction between intranasal instillation and oropharyngeal aspiration exposures that likely accounts for differences in effects and degree of effects; intranasal exposures are administered directly into the nasal passages, which includes a direct exposure to the brain via the olfactory tubercle, whereas oropharyngeal aspiration delivers a substance to the back of the throat to be inhaled into the lungs. Hence, the physiological effects of these exposure routes will demonstrate different toxicokinetic and, therefore, likely different effects.

Vitamin C is considered the primary reducing agent for Cr(VI), and it is an essential mineral for humans. Most animals can endogenously synthesize vitamin C in their livers, which reduces the translational significance of results from mouse or rat studies to human conditions. Guinea pigs are the only known rodent species that do not endogenously synthesize vitamin C, possibly making them a better rodent model for Cr(VI) toxicity. In addition to this physiological similarity with humans, Hartley guinea pigs are an outbred strain, which may better mimic human genetic diversity compared with inbred rodent strains [40]. To our knowledge, this is the first study to assess the effects of Cr(VI) on behavior and brain metal levels in a guinea pig model. Our data demonstrated Cr(VI) affected locomotor function, anxiety, memory, Cr hippocampal accumulation, and hippocampal essential metal homeostasis, suggesting neurotoxicity is an endpoint to be considered after human occupational exposures and that Cr(VI) neurotoxicity should be further investigated. The following sections will compare data generated in this guinea pig study with Cr(VI) neurotoxicity in other models.

### 4.1. Cr(VI) Induced Behavioral Effects and Brain Accumulation, Independent of Sex

We first considered the effects of Cr(VI) on behaviors in each assay with data pooled from both sexes. In this context, we observed a slight decrease in distance traveled in the open field assay by guinea pigs exposed to 0.4 mg/kg, though this effect was not significant and suggests this exposure has minimal effects on locomotion (Figure 2). We also observed a slight decrease in maximum speed by guinea pigs exposed to 0.4 mg/kg, though this effect is not significant (Figure 5). While locomotor effects were negligible in this study, other groups have reported impaired locomotion in rats and mice after exposure to greater Cr(VI) concentrations [11,12,24]. We recently reported impaired distance traveled in female Sprague–Dawley rats exposed to 0.05 mg/L Cr(VI) (sodium chromate) in drinking water for 8 weeks, though it is difficult to conclude if the inconsistency in locomotor effects results from a difference in species or exposure route [21,22].

In this same assay, guinea pigs exposed to 0.4 mg/kg exhibited slightly greater time spent frozen, suggesting anxiogenic effects (Figure 3) [31]. Compared to data obtained from the elevated plus maze, a standard assay for measuring anxiety, we only observed changes in exploration by guinea pigs exposed to 0.4 mg/kg. This study group exhibited decreased closed arm exploration and increased center area exploration (Figure 6). Decreased closed arm exploration suggests anxiolytic effects, but we observed little to no change in open arm exploration. This was initially puzzling; however, we considered the center area as a separate space and observed increased time in the center area, mirroring the decreased time in the closed arm. We interpreted these changes in the center area time as a manifestation of the rodent approach/avoidance conflict [32,41,42]. The approach/avoidance conflict correlates with center area exploration in the elevated plus maze with anxiogenic effects [41,43]. We suggest that guinea pigs in this group experienced an approach/avoidance conflict, resulting from Cr(VI)-induced anxiety. Evidence that guinea pigs may exhibit an approach/avoidance conflict in the elevated plus maze, alongside increased time freezing in the open field assay, suggests anxiogenic effects after exposure to 0.4 mg/kg [32].

In the Y-maze, Cr(VI) had little to no effect on spatial memory (Figure 7). However, in the novel object recognition test, Cr(VI) increased preference for the novel object in guinea pigs exposed to 0.2 mg/kg (Figure 8). This would appear to suggest Cr(VI) improved working memory, conflicting with previous reports. Estrela et al. reported impaired social memory in 2–3 month-old female Swiss mice exposed to 500 mL tannery effluent containing high levels of Cr(VI) (859 mg/L) for 2 h per day for 20 days and we previously reported impaired spatial memory in 18-month-old male Sprague-Dawley rats exposed to 0.1 mg Cr(VI)/L (sodium chromate) in drinking water after only 4 weeks exposure [22,23]. However, we also reported improved spatial memory in 7-month-old female rats in the Y-maze after 10 weeks of exposure to 0.1 mg Cr(VI)/L in drinking water (sodium chromate) [21]. In our previous study, we proposed that Cr(VI) induced significant hippocampal damage in female rats, leading to side preferences, and we believe hippocampal effects may result in increased novelty preference by guinea pigs in the present study. Here, we propose that Cr(VI) targets the guinea pig hippocampus to reduce competitive interference and improve memory consolidation between trials 1 and 2 of the novel object recognition test. At least one group reported hippocampal inactivation increased novel object preference when the assay was performed in a novel, impoverished environment [44]. Our study utilized a novel environment for this test, and guinea pigs performed each trial in the dark; thus, we propose Cr(VI) may target the hippocampus and decrease competitive inhibition to improve memory consolidation in this context. Further, Hegazy et al. reported increased preference for the novel object in adult male albino Wistar rats exposed to 0.5 mg/kg/day potassium dichromate by intranasal instillation for up to 2 months, which we will discuss in the context of our data later [11]. Data gathered from the Y-maze and novel object recognition test may appear to suggest memory impairments, and changes in novel object exploration may also be related to anxiogenic and altered exploratory behaviors we observed in the elevated plus maze and open field assay. Future studies will consider more rigorous assays to confirm the effects of Cr(VI) on working memory and provide more detailed analyses.

Considering Cr accumulation in this model, we observed increased Cr levels in the hippocampi of guinea pigs exposed to 0.2 or 0.4 mg/kg, with significantly increased amounts in those exposed to 0.8 mg/kg (Figure 9). These data are notable, as we previously only reported increased hippocampal Cr in geriatric female rats, whereas these guinea pigs were young animals [20].

### 4.2. Sex Differences in Cr(VI) Behavioral Effects

Considering sex differences in the open field assay, we observed a slight concentration-associated decrease in distance traveled by males, but we observed only a slight decrease in female travel distance in animals exposed to 0.4 mg/kg (Figure 2). Cr(VI) is reported to impair locomotor function in a variety of rodent models, but the majority of studies only considered males [11,12,24]. We previously reported that female rats exhibited decreased distance traveled in the open field assay after 8 weeks of exposure to low concentrations of Cr(VI) in drinking water (0.05 mg Cr(VI)/L, sodium chromate as the representative compound), but this effect was not apparent in males of that study [20,21]. While effects reported here are slight and exposure regimens were drastically different, there may be a species-specific effect on distance traveled.

Freezing behavior in the open field assay also varied between sexes. Cr(VI) had little effect on freezing episodes in male or female guinea pigs in the open field assay (Figure 3). When assessing total time frozen, we observed a slight increase in males exposed to 0.4 and 0.8 mg/kg but little to no change in females, suggesting trends in males were strong enough to drive the effect observed when pooling data from sexes. As males in these groups exhibited greater time frozen, some immobile for nearly half the assay, we suggest the decreased distance traveled by this group in the open field assay may be due to increased time frozen rather than locomotor impairments.

In the elevated plus maze, males exhibited little to no change in open arm exploration but decreased closed arm exploration after exposure to 0.4 mg/kg (Figure 6). These data suggest decreased anxiety without an increase in open arm exploration; however, we observed increased center area exploration across all male-exposed groups. As discussed above, we suggest that increased center area exploration is an anxiety response driven by the approach/avoidance conflict. Interestingly, females exhibited little to no change in center area exploration after Cr(VI) exposure; however, females exhibited decreased open arm exploration in 0.2 and 0.4 mg/kg exposed groups and increased open arm exploration in 0.8 mg/kg exposed guinea pigs (Figure 6). These data display a non-monotonic effect in which Cr(VI) exhibited anxiogenic effects in females exposed to 0.2 and 0.4 mg/kg, but anxiolytic effects in those exposed to 0.8 mg/kg. These data suggest a sex difference in guinea pig anxiety responses, where males undergo an approach/avoidance conflict while females exhibit greater anxiety. Compared to our previous study, which assessed open arm exploration in young rats (3-month-old at start of exposure) after a drinking water exposure, we observed a concentration-associated increase in anxiety by males and females [20,21]. Interestingly, we reported anxiolytic effects in 18-month-old males, similar to what we observed in young female guinea pigs exposed to 0.8 mg/kg [20]. Similarities between female guinea pigs in our highest exposed group and geriatric male rats from our previous study suggest Cr(VI) induces anxiogenic effects in some populations but anxiolytic effects in others, indicating more research is needed to fully understand the effects of Cr(VI) on anxiety.

We assessed spatial memory as non-alternating exploration in the Y-maze. During the 11th week of exposure, males from all exposure groups exhibited slightly increased non-alternations, but we observed a decrease in 0.2 mg/kg exposed females (Figure 7). These data suggest Cr(VI) impaired spatial memory in males but improved spatial memory in females exposed to low concentrations of Cr(VI); however, we believe decreasing non-alternating exploration in females may be a result of side preferences. We previously reported a concentration-associated increase in non-alternations by middle-aged (7-month-old) Sprague–Dawley female rats exposed to 0.05 and 0.1 mg Cr(VI)/L (sodium chromate) in drinking water for 10 weeks [21]. We attributed this change in rats to a side preference phenotype developed by significant hippocampal damage [45,46].

Cr(VI) had confusing effects on memory in the novel object recognition test during week 12 (Figure 8). Males exhibited increased preference for the novel object after exposure to 0.2 mg/kg. In females, we observed increased preference for the novel object in all exposed groups. At the moment, we do not have a clear answer for the variability of male guinea pig behavior in the novel object recognition test. Interestingly, Hegazy et al. reported significantly increased preference for novel objects in adult male albino Wistar rats after 1–2 months of exposure to 0.5 mg/kg/day potassium dichromate via intranasal instillation, but a decrease after exposure to 0.125 or 0.25 mg/kg/day Cr(VI) [11]. The weekly concentration used in that study is ~3x greater than the highest concentration used in our study (2.5 vs. 0.8 mg/kg/week, respectively); we observed increased preference for the novel object in nearly all exposure groups, while Hegazy et al. only observed an increased discrimination index in their highest exposure group (0.5 mg/kg/day). These data appear to suggest oropharyngeal aspiration delivers a more potent dose of Cr(VI) to rodents than intranasal instillation, possibly due to the complex nasal turbinates of rodents.

### 4.3. Cr(VI) Exposure Induced Hippocampal Cr Accumulation and Altered EssentialMetal Homeostasis

Accumulation of Cr in the hippocampus is only statistically significant for females exposed to 0.8 mg/kg, though we observed elevated hippocampal Cr in all exposed groups (Figure 9). Further analyses with essential metals reveal dyshomeostasis only in males—we observed decreased Na, but increased Zn (Figure 10, Table 2). Similar effects have been reported in other species (rats, mice, Japanese Quail, chickens, etc.), but data on regional accumulation remain sparse [5]. Quinteros et al. reported increased Cr accumulation in the hypothalamus and pituitary after a drinking water exposure to high concentrations of Cr(VI), though other brain regions were not assessed [19]. Ding et al. recently published Cr accumulation across 10 brain regions after daily intraperitoneal injection with Cr(VI) for 14 or 28 days [18]. This study reported significantly increased Cr across all regions assessed, with the greatest Cr accumulation in the thalamus and hypothalamus. We recently published results for regional brain Cr accumulation in rats after a 90-day exposure to Cr(VI) in drinking water, and observed that the hippocampus was the major brain region to accumulate Cr [20]. Notably, we only observed hippocampal Cr accumulation in the female hippocampus. While hippocampal Cr accumulation appears consistent across these studies, only three other studies reported essential metal dyshomeostasis. Our drinking water study demonstrated that Cr(VI) altered nearly every essential metal in the hippocampus, even in tissues that did not accumulate Cr, and with distinct age and sex differences. Two other studies reported essential metal dyshomeostasis in the brain after Cr(VI) exposure, one study reported increased Mn, but decreased Cu, Fe, and Zn after intraperitoneal injection of potassium dichromate in male Swiss albino mice; while the other reported decreased Ca, Fe, and Zn, but increased Mn, in chickens after 42 days exposure to potassium dichromate by oral gavage [47,48]. While changes in brain essential metal levels appear to be distinct across species, exposure route, and brain region, these data altogether suggest essential metal dyshomeostasis may be a critical step in Cr(VI) neurotoxicity.

## 5. Conclusions

We demonstrate that 9–12 weeks of exposure to occupationally relevant concentrations of Cr(VI) induced behavioral effects in male and female Hartley guinea pigs. We observed Cr(VI) exposure impaired locomotor function, altered anxiety states, and altered memory consolidation and recall, with sex differences across neurobehaviors. Further, our data indicate that Cr accumulated in the hippocampus and induced essential metal dyshomeostasis in the male hippocampus, suggesting that occupational exposure to low concentrations of Cr(VI) induces behavioral neurotoxicity, alters brain metal levels, and likely damages the hippocampus. Further, these data affirm that a guinea pig oropharyngeal aspiration model is a viable alternative to other rodent models for Cr(VI) toxicity testing.

## Figures and Tables

**Table 1.** Behavior Assay Schedule and Endpoints.

Week (s)	Behavior Assay	Behaviors Assessed
1–8	None	None
9	Open Field Assay	Distance Traveled, Center Area Exploration, Freezing Behavior, Maximum Speed Attained
10	Elevated Plus Maze	Open Arm Exploration, Closed Arm Exploration, Center Area Exploration
11	Y-Maze	Non-Alternating Exploration
12	Novel Object Recognition Test	Object Preference
13	None	None
14	Guinea Pigs Sacrificed	

**Table 2.** Zinc Chromate altered Na, Ni, and Zn levels in the male hippocampus. Data represent mean SEM (ng/g).

Essential Metal	Control	0.2 mg/kg	0.4 mg/kg	0.8 mg/kg
Na	1,101,711±20,567	1,029,860*±24,034	1,093,800±50,720	1,028,535±93,390
Mg	120,009±1858	118,505±743	127,945±4783	114,199±9566
K	2,866,768±60,874	2,842,675±28,756	3,037,700±107,072	2,710,901±227,753
Ca	42,589±880	41,233±663	45,102±2493	40,572±3681
Mn	288.2±5.693	285.6±6.533	311.5±14.07	281.7±22.13
Fe	15,771±954	16,163±847	16,187±1190	15,390±1713
Co	6.242±0.187	6.320±0.096	6.635±0.281	6.200±0.532
Cu	2170±43	2088±56	2154±74	2068±189
Zn	12,839±222	12,185±165	13,608*±588	12,297±973
Se	224.7±5.798	226.5±9.786	218.1±6.226	212.0±20.88

* *p* < 0.05, in bold.

## Data Availability

Data are contained here in the manuscript or available upon request.
